# Narcotism in Infancy

**Published:** 1882-11

**Authors:** 


### Selections.

### Narcotism in Infancy.

Dr. H. Cripps Lawrence says in the *Practitioner:*

Given a healthy child to start with certain results will follow the administration of narcotics, with an equally certain regularity.

1. In the early administration of a narcotic, more or less diversified, according to the temperament of the infant, a modified degree of excitement will supervene, to be followed by sleep; and this favorable condition of things may recur for a period varying from a few days to a week or two. If, however, the repetition of the narcotic be at all frequent, or if it be exhibited in any but a very small amount, this period of favorable narcosis will be proportionately diminished, and the chances of its lethal effect increased.

2. In some infants the effects of the narcotic will be to speedily constipate, for, as in the adult, so in the infant, a narcotic lessens at once the secretions and movements of the stomach and intestines, and consequently digestion becomes arrested.

There is, however, a class of cases met with in infancy, wherein infants, the subject of imperfect digestion associated with colic, become for a time benefited by a moderate amount of narcotic. Here the sedative acts by checking peristalsis, and so enables the food to remain a longer time in contact with the digestive juices, and thus to become more perfectly digested. This condition, however, is more frequent in early childhood than in infancy.

A third and more frequent sign of the administration of a narcotic in the infant is the occurrence, sometimes the concurrence, of diarrhœa and vomiting. When these occur a longer time will elapse than when such conditions are absent, before profound narcosis ensues.

3. In addition to the above, diaphoresis and diuresis may occur, one or both. Either of these may and often will be present in an inverse proportion to the other. The predominance of either will be dependent upon the amount of fluid ingesta, the character of the fluids taken, and the condition of the temperature of the weather present at the time. Obviously, warm weather or an over-heated atmosphere will promote diaphoresis, while cold weather would augment diuresis.

4. The intestinal evacuations soon alter in character, becoming paler and more solid in consistency, diminished in their frequency, and increasingly more and more deficient in bile, accompanied by a change in color, from yellowish-green to verdant green, with or without mucus, and if very constipated, accompanied by streaks of blood, owing to the tenesmus which is associated with the expulsion of the hardened, ill-digested fæces.

5. Another vital symptom, though of later occurrence than the preceding, is marked depression of the vital powers, evidenced by sinking of the anterior fontanelle. This is accompanied by other signs of want of vitality, viz., feebleness of pulse, loss of weight, an earthy complexion, wasting, especially marked in the pinched and anxious expression of the face. By this time the infant alternates between efforts at peevish whimpering and imperfect somnolency.

The pupils will be found, in the earlier stages of narcotism in infancy, to be frequently contracted, but when the stage of fontanelle depression is reached they may contract or dilate, and one pupil may be larger than the other. This condition I believe to be dependent upon variations in the amount of fluid present in the ventricles of the brain.

So soon as this stage is reached, any subsequent dose of a narcotic may prove speedily lethal, and the greatest care and caution become necessary to avert an outward result. It is at this point that I believe the cumulative effect and action of the narcotic assumes a point of much peril to the infant. One of two results may now supervene: either clonic convulsions may set in, though these are not very frequent, or, and much more commonly, profound and perhaps fatal coma.—*Medical and Surgical Reporter.*

